# The effects of the diagnosis-intervention packet payment reform in China: evidence from Guangzhou

**DOI:** 10.3389/fpubh.2025.1633222

**Published:** 2025-09-05

**Authors:** Yuhao Wang, Xiaoqing Huang, Nana Lu, Xin Xu, Lina Wang, Wenwen Du, Wei Xu

**Affiliations:** ^1^School of International Pharmaceutical Business, China Pharmaceutical University, Nanjing, China; ^2^Zhongda Hospital, Southeast University, Nanjing, China

**Keywords:** DIP payment reform, inpatient costs, out-of-pocket payments, interrupted time series analysis, policy evaluation

## Abstract

**Background:**

This study evaluated the impact of Guangzhou’s Diagnosis-Intervention Packet (DIP) payment reform, introduced as an alternative to the traditional fee-for-service (FFS) model, on inpatient costs, patients’ out-of-pocket (OOP) payments, hospital length of stay (LOS) and 30-day readmission rate.

**Method:**

We conducted a single-group interrupted time series analysis using monthly data from the Guangzhou Urban Employee Basic Medical Insurance Scheme, spanning January 2017 to June 2020. Outcome indicators included inpatient cost per case, medication expenditures, medical consumables expenditures, diagnostic and therapeutic service expenditures, OOP payments, LOS and 30-day readmission rate.

**Results:**

While the DIP reform was associated with a modest reduction in the rate of LOS decline, it did not significantly lower inpatient cost. Instead, total inpatient expenditures exhibited a sustained upward trend in the post-reform period. Moreover, OOP payments per case increased significantly following the reform, indicating a heavier financial burden on patients. Sub-item analysis revealed that medication expenditures declined and stabilized after a pre-policy drop, whereas diagnostic and therapeutic service expenditures continued to grow without evident signs of deceleration. The 30-day readmission rate remained largely stable throughout the study period.

**Conclusion:**

The DIP reform in Guangzhou did not achieve its intended goals of reducing inpatient expenditures or alleviating patients’ financial burdens. These unintended effects were driven by the incentive structure of the DIP system—particularly its reliance on historical cost data for RW calculation and its reimbursement deduction method—which encouraged hospitals to shift costs and elevate OOP payments. Policymakers should reassess DIP’s algorithmic parameters and strengthen cost-accounting transparency to ensure more equitable and efficient medical insurance fund allocation.

## 1 Introduction

### 1.1 Background of the diagnosis-intervention packet (DIP) payment reform in China

Medical insurance payment system reform is one of the crucial measures for controlling irrational medical practices (1). The payment systems can be categorized into retrospective and prospective payment systems. The fee-for-service (FFS) payment method is the most traditional retrospective payment method (2). However, it is prone to moral risks such as overtreatment due to information asymmetry and provider-induced demand (3–5).

Prospective payment systems encompass capitation, per-diem, and diagnosis-related group (DRG) payment systems (6–8). Among these, the DRG payment is a globally recognized approach that ensures the quality of medical care while facilitating better cost control (9, 10). Originating in the United States, the DRG payment groups patients into different diagnostic groups based on age, sex, number of days in the hospital, clinical diagnosis, severity of illness, comorbidities, and complications. The medical insurance department calculates the standard payment for each DRG and reimburses hospitals accordingly (11, 12). Compared to FFS, DRG payment contributes more effectively to reducing unreasonable medical expenditures, improving the efficiency of medical insurance fund utilization, and enhancing the management capabilities of hospitals. However, this approach also carries the risk of patient disincentives and reduced quality of care (13, 14).

**Figure 1:**
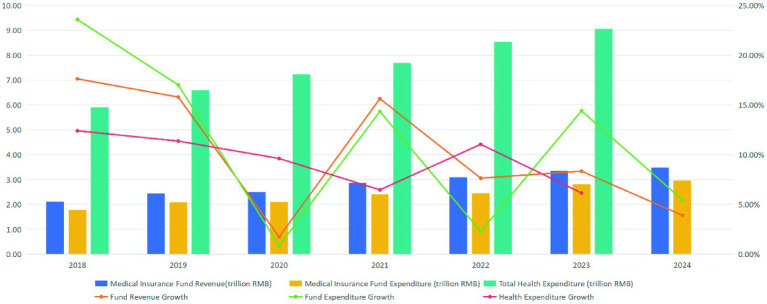
Trends in medical insurance fund revenue, expenditure, and total health expenditure in China (2018–2024).

Since the establishment of the National Healthcare Security Administration (NHSA) in 2018, China’s medical insurance fund has grown steadily in both scale and function, as illustrated in Figure 1. Between 2018 and 2024, medical insurance fund revenue increased from 2.11 trillion RMB to 3.48 trillion RMB, representing a CAGR of 8.71%. Over the same period, medical insurance fund expenditure rose from 1.78 trillion RMB to 2.97 trillion RMB, with a slightly higher CAGR of 8.95%. While both revenue and expenditure have expanded significantly, the persistent faster growth of expenditure—evident in specific years such as 2020, 2022, and 2023—signals escalating fiscal pressure. This pressure is further compounded by the increase in total health expenditure, which rose from 5.91 trillion RMB in 2018 to 9.06 trillion RMB in 2023, with a CAGR of 8.99%, reflecting strong and sustained demand for health financing. Facing growing fiscal pressure, the NHSA prioritized payment system reform as a critical strategy to standardize medical practices and, on that basis, reduce unnecessary expenditures and strengthen the sustainability of the healthcare financing system. In 2021, the NHSA issued the *Three-Year Action Plan for DRG/DIP Payment Reform*, aiming to extend DRG or DIP-based payment models to all eligible inpatient care institutions by the end of 2025.

Diagnosis-Intervention Packet (DIP) is a prospective payment system independently developed by the NHSA, drawing inspiration from the DRG payment system. The DIP payment system utilizes mathematical models to analyze and categorize disease data, diagnostic procedures, and treatment methods comprehensively, enabling efficient management and reimbursement grouping (15). Its approach, features, and payment model are similar to those of the DRG payment. However, a key distinction lies in their grouping methodologies: while the DRG payment relies on clinical experience as its foundation through expert judgment and selection of clinical pathways for “one group of multiple diseases” or “one group of multiple operations,” the DIP payment emphasizes statistical analysis of objective real-world data by exhaustively clustering disease diagnoses and surgical procedures from historical cases into groups characterized by “one group of one operation for one disease” (16–18).

As China deepens its healthcare reforms, especially in payment system restructuring, evaluating the effectiveness of newly implemented mechanisms such as DIP is both timely and policy-relevant. Guangzhou, the capital city of Guangdong Province, is renowned for its robust economy with the fourth-largest gross domestic product (GDP) in China. Importantly, it was also among the first to implement the DIP payment system, launching the reform in January 2018. This study aims to evaluate whether the DIP payment reform has achieved its intended effects on inpatient medical service delivery. Specifically, it examines the reform’s impact on three key dimensions: (1) the average inpatient cost per admission, (2) the length of hospital stay (LOS) and 30-day readmission rate, and (3) patients’ out-of-pocket (OOP) payments. While the primary objective is to assess the effectiveness of the DIP model in controlling medical costs and alleviating patient financial burden, the study also extends its inquiry to the internal logic and structural design of the DIP system—particularly in the event that the expected policy outcomes are not realized. By integrating empirical evaluation with a mechanism-based analysis, this research aims to offer a more comprehensive understanding of how payment system design shapes implementation outcomes and informs future policy refinement.

### 1.2 The operational framework of the DIP payment system

**Figure 2:**
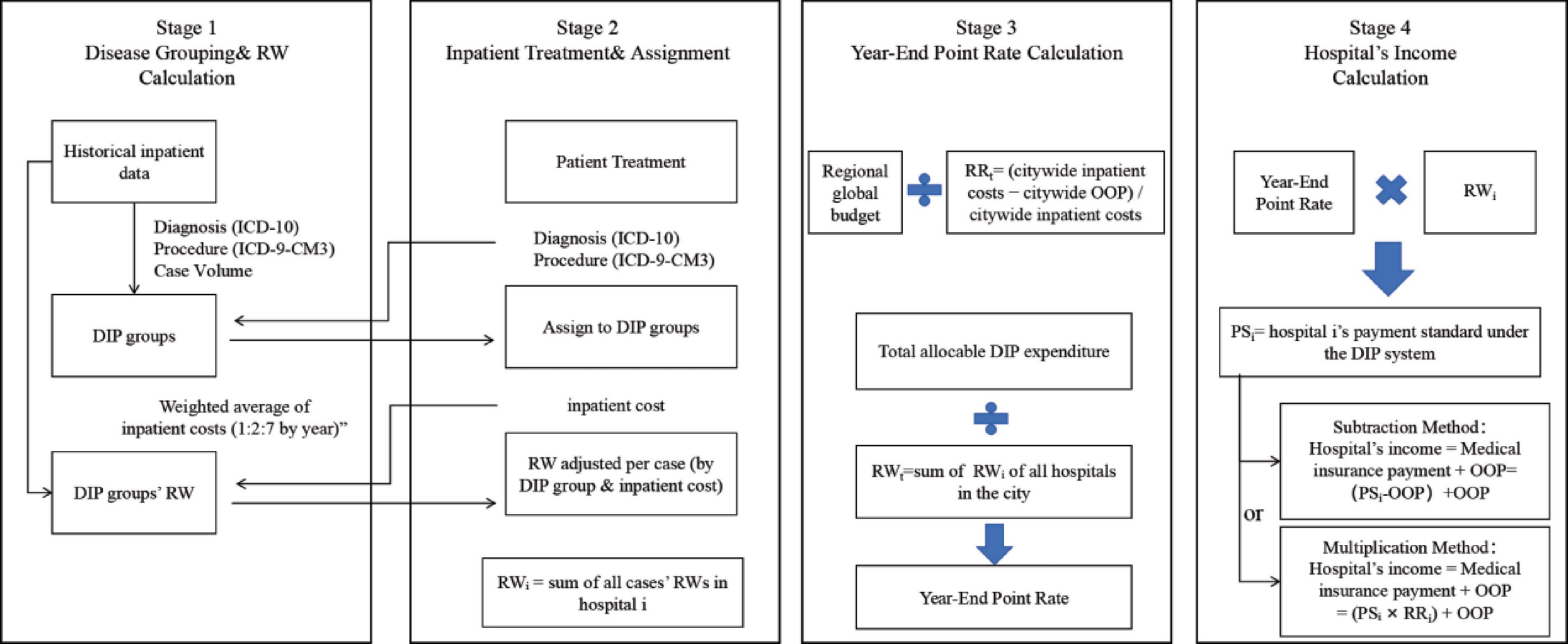
The framework of the DIP payment reform in China.

The DIP payment system in China operates through four sequential stages, as illustrated in Figure 2.

Stage 1 involves disease grouping and relative weight (RW) calculation. The medical insurance authority utilizes historical inpatient data from the previous three years to formulate DIP groups based on primary diagnoses (ICD-10), procedures (ICD-9-CM-3), and case volume thresholds. Each group is then assigned a RW, calculated as the weighted average of inpatient costs across these three years *(using a 1:2:7 weighting for years T − 3, T − 2, and T − 1, respectively)*.

Stage 2 covers inpatient admission and RW assignment. During the current year, newly admitted patients are mapped to pre-defined DIP groups based on their principal diagnosis and procedures at discharge, coded using ICD-10 and ICD-9-CM-3. If a patient’s inpatient cost falls within the defined cost range for the group (e.g., 0.5 to 2 times the historical weighted average in Guangzhou), the standard RW is applied; otherwise, the RW is adjusted according to local outlier policies. RWᵢ represents the sum of all individual case RWs for patients treated at hospital *i*.

Although medical insurance funds are typically disbursed on a monthly basis according to each hospital’s accumulated RWᵢ from the preceding month, the final reimbursement is determined through a year-end adjustment that accounts for the hospital’s total service provision over the entire fiscal year. Therefore, this study focuses on the year-end settlement process and does not address monthly payment mechanisms. In this context, Stage 3 involves the determination of the year-end point rate. The regional medical insurance budget is first divided by RRₜ (the proportion of total inpatient costs reimbursed by the insurance fund, calculated as total inpatient cost minus total OOP payments divided by total inpatient costs), resulting in the total distributable expenditure. This amount is then divided by RWₜ (the sum of RWᵢ across all designated hospitals) to derive the year-end point rate used for final settlement.

Stage 4 covers the calculation of each hospital’s income under the DIP system. A hospital’s payment standard (PSᵢ) is calculated by multiplying its RWᵢ by the year-end point rate. Since PSᵢ is calculated based on the total expenditure benchmark rather than the insurance-funded benchmark, the medical insurance authority deducts the non-reimbursable portion—namely, the OOP payments—from PSᵢ when determining the amount actually payable by the fund.

In China, two methods are commonly applied to implement this deduction: the subtraction method, which subtracts the actual OOP amount from PSᵢ, and the multiplication method, which applies the hospital’s reimbursement ratio to PSᵢ to estimate the reimbursable share. This amount represents the portion covered by the insurance fund. This insurance-covered amount, when combined with the OOP payments collected directly from patients, constitutes the hospital’s total income under the DIP system. If this total income exceeds the hospital’s total inpatient costs for all discharged cases, the institution is deemed to have operated at a “surplus” under the DIP system; conversely, if the income falls short, the hospital is considered to have incurred a “deficit.” Through this surplus-deficit mechanism, the medical insurance authorities aim to incentivize cost containment and promote reductions in overall inpatient costs.

**Figure 3:**
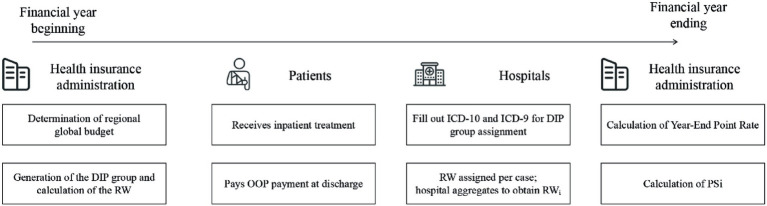
The financial flow of the DIP payment reform in China.

Figure 3 illustrates the financial flow under the DIP payment system. Initially, the healthcare security administration sets the regional global budget and defines the DIP group classification and corresponding base RWs. Upon discharge, patients pay OOP expenses under the FFS scheme. Hospitals then submit diagnostic and procedural codes, which are used to assign each case to a DIP group and determine its RW. Throughout the year, each hospital accumulates its RW_i_ by summing the RWs of all inpatient cases. At the end of the year, the administration calculates the Year-End Point Rate and the PS_i_ for each hospital—steps detailed in Figure 2. The final insurance payment amount is then computed using either a subtraction or multiplication method, as specified by regional policy design.

## 2 Materials and methods

### 2.1 Sample data

#### 2.1.1 Data source

We obtained data from the Guangzhou Urban Employee Basic Medical Insurance Scheme database, provided by the Guangzhou Healthcare Security Administration, covering the period from January 2017 to June 2020. The dataset included patient age; sex; admission and discharge date; diagnosis, procedure and cost; and hospital level (tertiary, secondary, or primary).

### 2.2 Indicators

**Figure 4:**
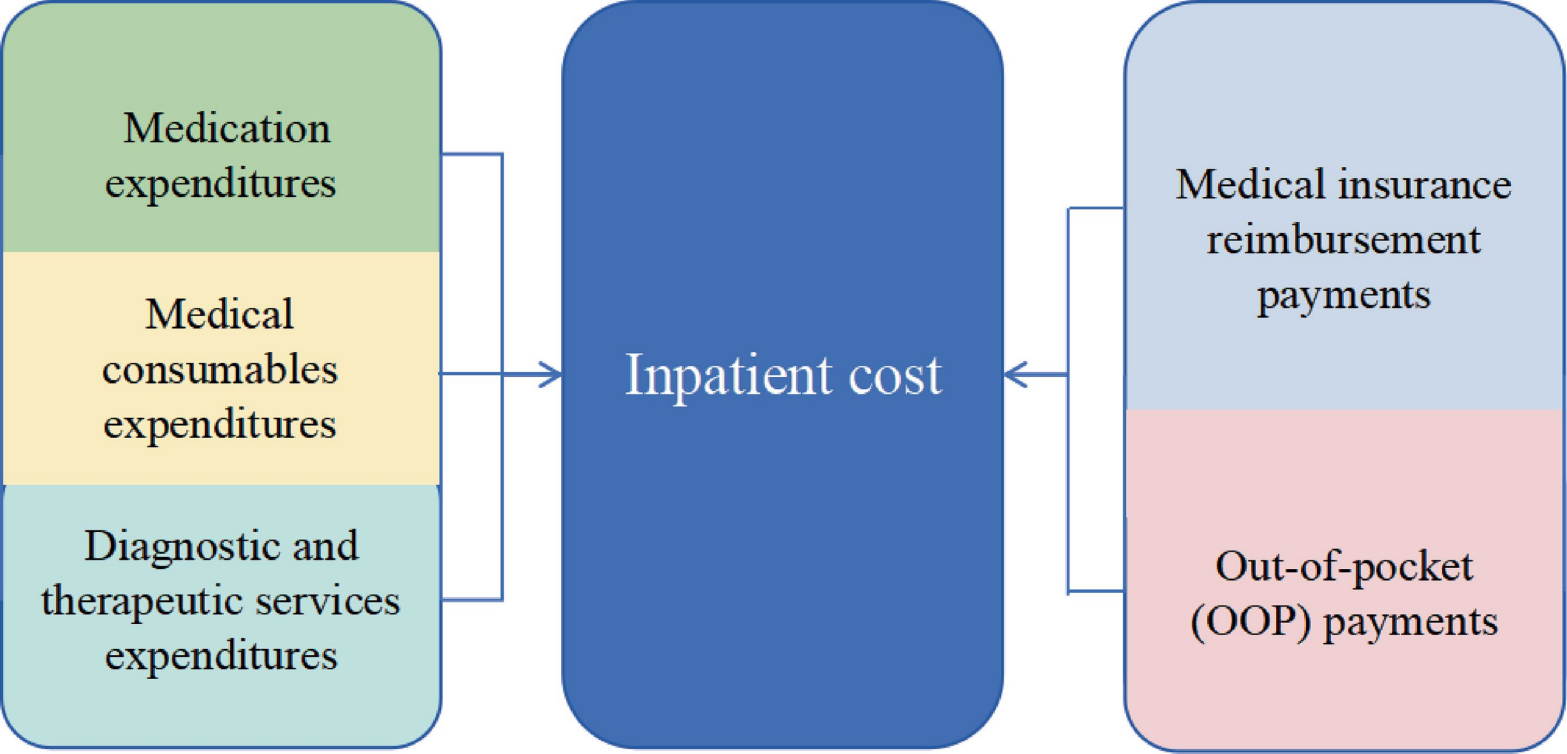
Composition and financing structure of inpatient cost.

We employed a set of outcome indicators categorized into three analytical dimensions to evaluate the impact of the DIP payment reform. First, in the dimension of inpatient medical expenditures, we examined the inpatient cost per case and further decomposed it into three components—medication expenditures, medical consumables expenditures, and diagnostic and therapeutic service expenditures—to capture changes in expenditure composition. Second, in the dimension of patient financial burden, we examined OOP payments per case, representing the share of hospitalization costs directly borne by patients (see Figure 4 for the structural relationship between cost components). Third, in the dimension of hospital service efficiency, we included LOS, defined as the number of inpatient days per admission, as an indicator of resource utilization. Additionally, we included the 30-day readmission rate to reflect short-term quality of care and treatment outcomes. To account for the effect of inflation, all expenditure-related variables were adjusted to 2017 constant prices using the Urban Residents Consumer Price Indices by Health Care, as published by the Guangzhou Statistics Bureau. Annual CPI values relative to the previous year were 106.1 in 2018, 103.8 in 2019, and 100.9 in 2020. Cumulative CPI multipliers were calculated to convert all values to 2017-based real terms. Subsequently, natural logarithmic transformations were applied to stabilize variance and improve comparability.

### 2.3 Methods

We used single-group interrupted time series analysis to compare the changes in various indicators before and after the implementation of the DIP payment reform (19–21). We constructed interrupted time series models in months, with January 2018 as the policy intervention month, involving 42 time points in three years from January 2017 to June 2020, including 12 months before and 30 months after the intervention. The single-group interrupted time series model is as follows.

$$Y_{t}=\beta_{0}+\beta_{1}T_{t}+\beta_{2}X_{t}+\beta_{3}T_{t}X_{t}+\varepsilon_{t.}$$

In this model, $Y_{t}$ represents the indicators of interest in this paper for each month, and $T_{t}$ is the monthly linear time trend over 42 months. $X_{t}$ is the policy intervention variable, with January 2018 serving as the boundary. The 12 months prior to the intervention were assigned a value of 0, and the 30 months following the intervention were assigned a value of 1. $T_{t}X_{t}$ is a continuous variable indicating the time variable after the implementation of the DIP payment reform, with a value of 0 before the intervention and a value of 1 after the intervention.

$\beta_{0}$ represents the baseline level of the indicators, $\beta_{1}$ reflects the monthly slope of each indicator before the implementation of the DIP payment reform, and $\beta_{2}$ represents the change at the first point after the implementation of the DIP payment reform (i.e., January 2018), indicating the short-term effect of the policy. $\beta_{3}$ represents the change in the monthly trend of the outcome indicator following the implementation of the DIP payment reform, relative to the pre-intervention slope, reflecting the long-term effect of the policy. To account for potential autocorrelation in the time series data, we employed the Prais-Winsten estimation method with robust standard errors and assessed model adequacy using the Durbin-Watson statistic. Additionally, we conducted the Ljung-Box tests on the residuals to examine whether they satisfied the white noise assumption.

## 3 Results

### 3.1 Descriptive statistics of the study dataset

Table 1 summarizes the descriptive characteristics of inpatient cases included in this study. A total of 472,958 admissions were recorded during the pre-reform period (January to December 2017), and 1,841,989 during the post-reform period (January 2018 to June 2020). The proportion of male patients remained relatively stable, accounting for 45.48% before the reform and 46.91% after. The average age of inpatients decreased from 66.87 years (SD = 16.66) to 59.65 years (SD = 18.60), and the mean length of stay declined from 10.09 days (SD = 10.12) to 9.50 days (SD = 9.79). The 30-day readmission rate was 5.37% before the reform and 4.61% after the reform. The average total inpatient cost was 15,320.16 RMB (SD = 23,143.62) in the pre-reform period and increased to 16,973.84 RMB (SD = 25,608.85) in the post-reform period. Among its components, medication expenditures decreased from 4,965.40 RMB (SD = 9,324.10) to 4,320.24 RMB (SD = 7,673.69), medical consumables expenditures increased from 2,925.69 RMB (SD = 10,032.37) to 3,629.66 RMB (SD = 11,710.53), and diagnostic and therapeutic service expenditures increased from 7,429.07 RMB (SD = 9,534.62) to 9,023.94 RMB (SD = 11,416.40). Additionally, out-of-pocket (OOP) payments per case rose from 3,388.28 RMB (SD = 5,972.84) to 4,216.85 RMB (SD = 7,673.69), indicating a potential increase in patient financial burden. Based on this dataset, we will conduct further empirical analysis to assess the causal impact of the DIP reform.

**Table 1:** Details of the sample data.

Variable	Before reform (January to December 2017)	After reform (January 2018 to June 2020)
Sample size, *n*	472,958	1,841,989
Male, *n* (%)	215,092 (45.48%)	864,012 (46.91%)
Age, mean (SD)	66.87 (16.66)	59.65 (18.60)
Length of stay (days), mean (SD)	10.09 (10.12)	9.50 (9.79)
30-day readmission rate	5.37%	4.61%
Inpatient cost, mean (SD) [RMB]	15,320.16 (23,143.62)	16,973.84 (25,608.85)
Medication expenditures, mean (SD) [RMB]	4,965.40 (9,324.10)	4,320.24 (7,673.69)
Medical consumables expenditures, mean (SD) [RMB]	2,925.69 (10,032.37)	3,629.66 (11,710.53)
Diagnostic and therapeutic services expenditures, mean (SD) [RMB]	7,429.07 (9,534.62)	9,023.94 (11,416.40)
OOP payments, mean (SD) [RMB]	3,388.28 (5,972.84)	4,216.85 (7,673.69)

SD, standard error; OOP, out-of-pocket.

### 3.2 Results of ITSA

#### 3.2.1 Changes in the inpatient cost per case

**Figure 5:**
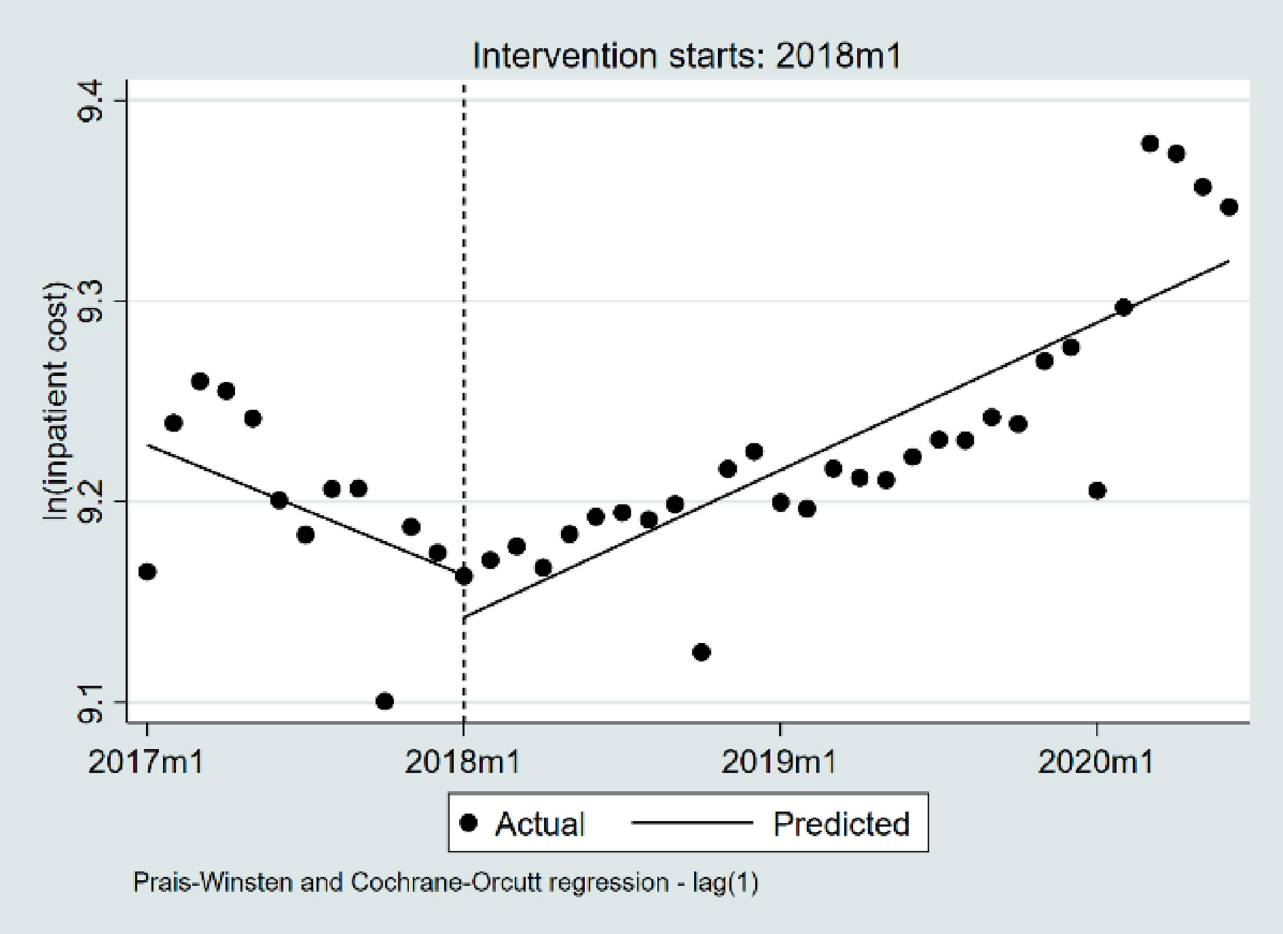
The changes in the inpatient cost per case 2017 m1, January 2017; 2018 m1, January 2018; 2019 m1, January 2019; 2020 m1, January 2020.

The changes in inpatient cost per case in the study region based on logarithmic transformation, are illustrated in Table 2 and Figure 5. At the beginning of the observation period, the baseline level of inpatient cost per case was estimated to be 9.23 on the natural logarithmic scale (*p* < 0.001), corresponding to approximately 10,198 RMB after exponentiation. During the pre-DIP period, the monthly trend showed a nonsignificant decline in inpatient cost (*β*_1_ = −0.0054, *p* = 0.317), representing an average monthly decrease of approximately 0.54%. At the point of DIP policy implementation, the immediate level change was also not statistically significant (*β*_2_ = −0.0210, *p* = 0.558), corresponding to a relative decrease of approximately 2.1%. During the post-DIP period, the monthly slope significantly increased (*β*_3_ = 0.0115, *p* = 0.044), indicating a continued monthly growth of 1.16% in inpatient cost following policy implementation.

**Table 2:** The changes in the inpatient cost per case.

Coefficient	Estimate	Standard error	*t*	*P*	95% confidence interval	Durbin-Watson statistic	Ljung-Box *p*-value
*β*_1_	−0.01	0.01	−1.01	0.317	−0.02, 0.01	1.86	0.871
*β*_2_	−0.02	0.04	−0.59	0.558	−0.09, 0.05		
*β*_3_	0.01	0.01	2.08	0.044	0.00, 0.02		
*β*_0_	9.23	0.04	255.63	0.000	9.16, 9.30		

#### 3.2.2 Changes in the medication expenditures per case

**Figure 6:**
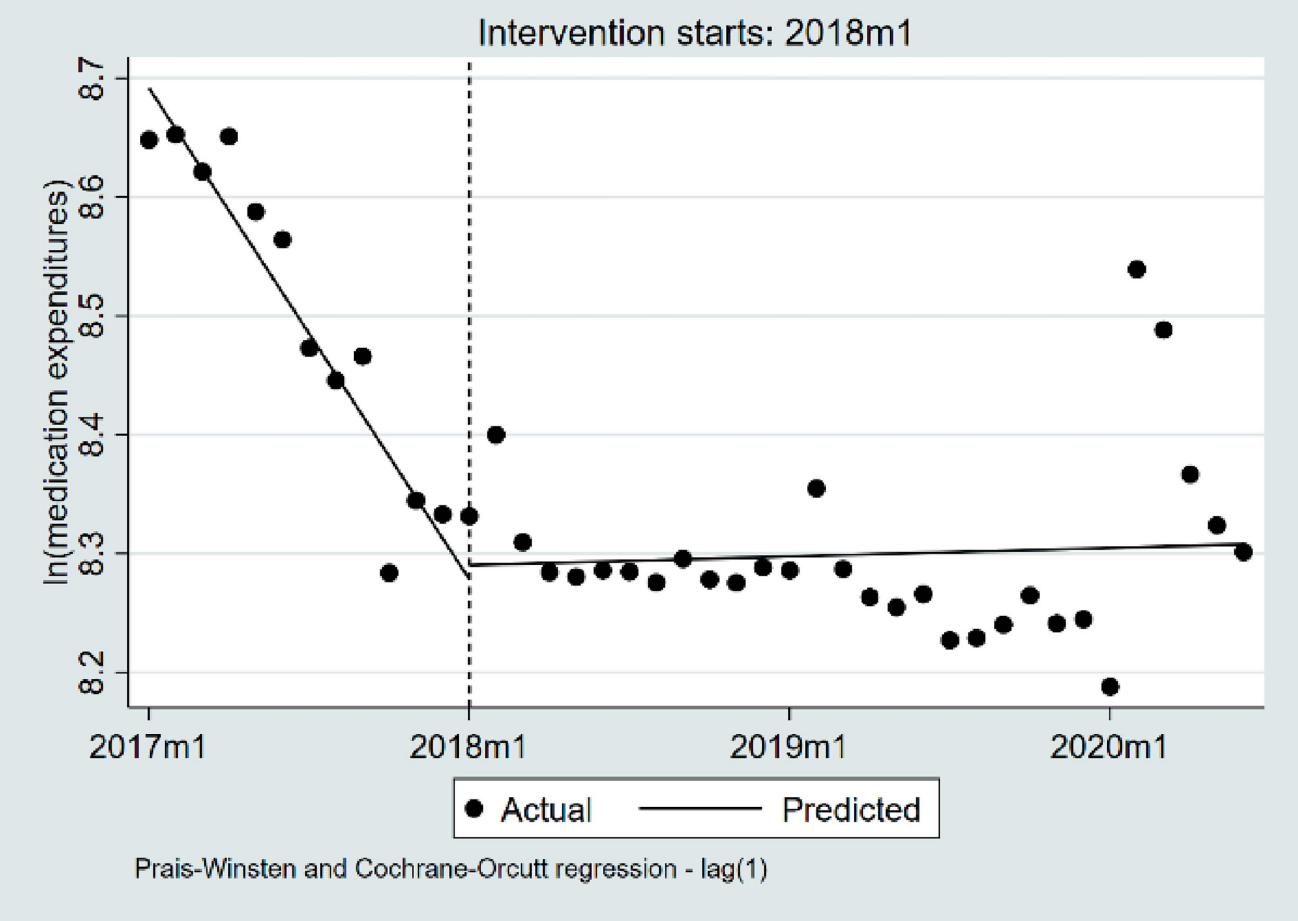
The changes in the medication expenditures per case. 2017 m1, January 2017; 2018 m1, January 2018; 2019 m1, January 2019; 2020 m1, January 2020.

We summarize the changes in medication expenditures per case based on a natural logarithmic transformation in Table 3 and Figure 6. At the outset of the observation period, the baseline level of medication expenditures per case was estimated at 8.69 on the natural logarithmic scale (*p* < 0.001), corresponding to approximately 5,944 RMB after exponentiation. During the pre-DIP period, medication expenditures showed a statistically significant monthly decline of approximately 3.38% (*β*₁ = −0.0344, *p* < 0.001). The implementation of the DIP policy was associated with a nonsignificant immediate level increase of approximately 1.21% (*β*₂ = 0.0120, *p* = 0.808). Following the intervention, the monthly trend exhibited a significant upward shift of approximately 3.57% relative to the pre-policy slope (*β*₃ = 0.0351, *p* < 0.001), indicating that the post-DIP trend reversed the prior decline and shifted toward a steady increase in medication expenditures.

**Table 3:** The changes in the medication expenditures per case.

Coefficient	Estimate	Standard error	*t*	*P*	95% confidence interval	Durbin-Watson statistic	Ljung–Box *P*-value
*β*_1_	−0.03	0.01	−6.23	0.000	−0.05, −0.02	1.92	0.522
*β*_2_	0.01	0.05	0.24	0.808	−0.09, 0.11		
*β*_3_	0.04	0.01	5.50	0.000	0.02, 0.05		
*β*_0_	8.69	0.03	295.78	0.000	8.63, 8.75		

#### 3.2.3 Changes in the medical consumables expenditures per case

**Figure 7:**
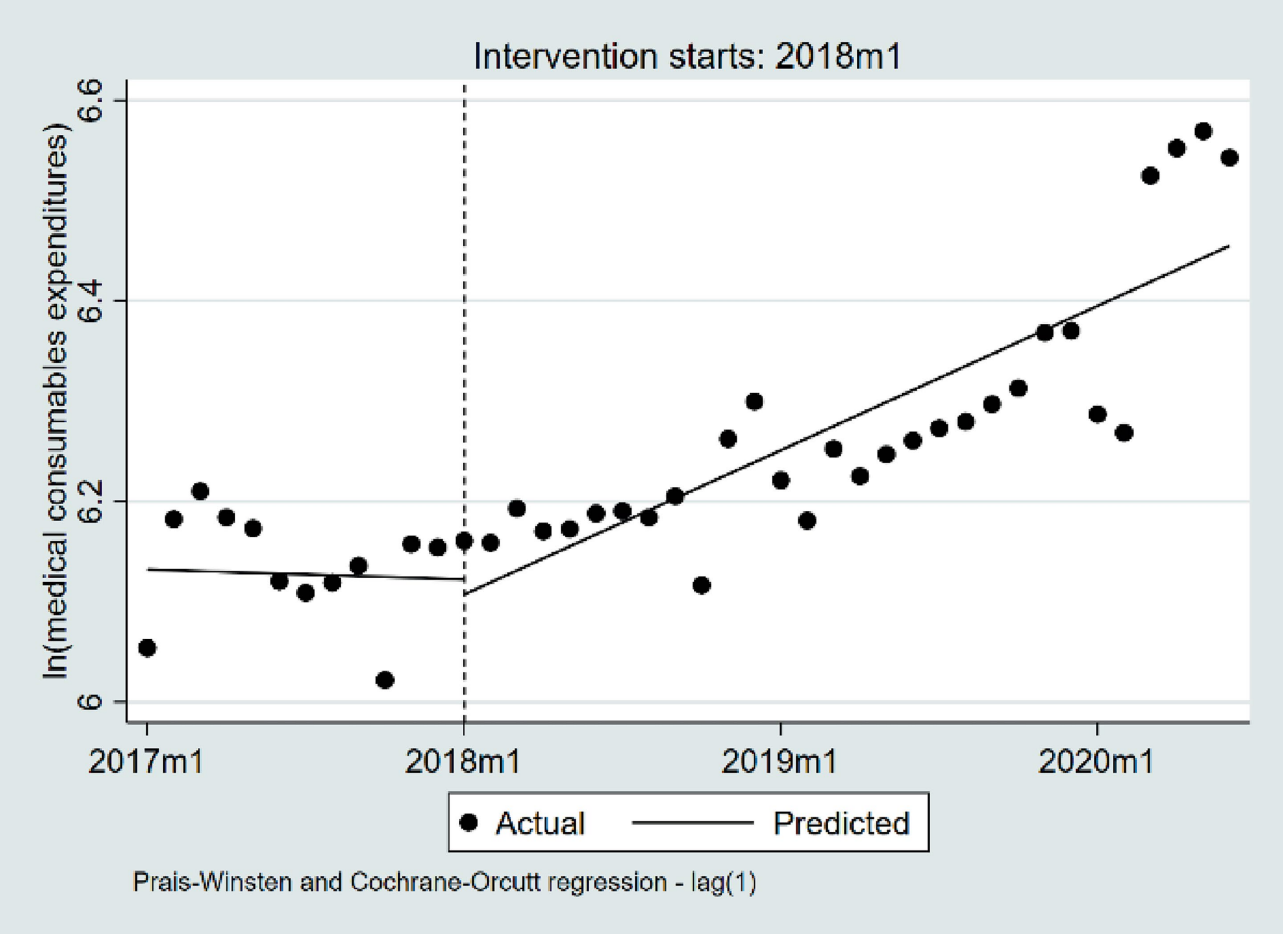
The changes in the medical consumables expenditures per case. 2017 m1, January 2017; 2018 m1, January 2018; 2019 m1, January 2019; 2020 m1, January 2020.

We summarize the changes in medical consumables expenditures per case, based on a natural logarithmic transformation, in Table 4 and Figure 7. At the beginning of the observation period, the baseline level of medical consumables expenditures per case was estimated at 6.13 on the natural logarithmic scale (*p* < 0.001), corresponding to approximately 462 RMB after exponentiation. During the pre-DIP period, medical consumables expenditures exhibited a virtually flat trend, with a statistically non-significant monthly change of approximately −0.05% (*β*_1_ = −0.0005, *p* = 0.950). The introduction of the DIP policy did not lead to a significant immediate level change, as reflected by a negligible relative decrease of 6.85% (*β*_2_ = −0.071, *p* = 0.204). In the post-DIP period, the monthly trend exhibited a mild upward shift relative to the pre-policy slope (*β*_3_ = 0.0106, *p* = 0.254), corresponding to a potential monthly increase of 1.07%, although this change was not statistical significance.

**Table 4:** The changes in the medical consumables expenditures per case.

Coefficient	Estimate	Standard error	*t*	*P*	95% confidence interval	Durbin-Watson statistic	Ljung–Box *P*-value
*β*_1_	0.00	0.01	−0.06	0.950	−0.02, 0.02	1.80	0.535
*β*_2_	−0.07	0.05	−1.29	0.204	−0.18, 0.04		
*β*_3_	0.01	0.01	1.16	0.254	−0.01, 0.03		
*β*_0_	6.13	0.06	105.70	0.000	6.01, 6.25		

#### 3.2.4 Changes in the diagnostic and therapeutic service expenditures per case

**Figure 8:**
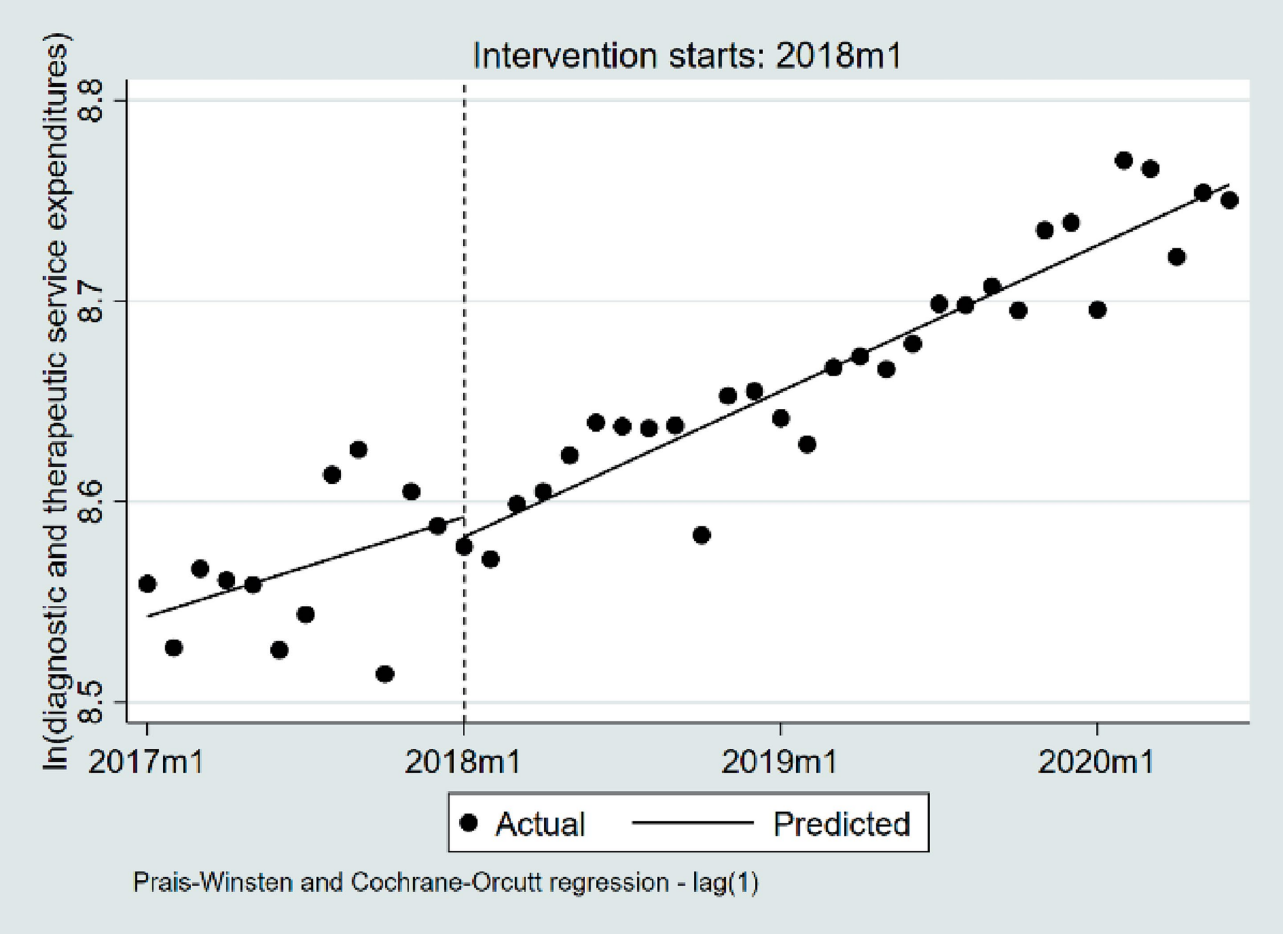
The changes in the diagnostic and therapeutic services expenditures per case. 2017 m1, January 2017; 2018 m1, January 2018; 2019 m1, January 2019; 2020 m1, January 2020.

We summarize the changes in diagnostic and therapeutic service expenditures per case, based on a natural logarithmic transformation, in Table 5 and Figure 8. At the beginning of the observation period, the baseline level was estimated at 8.54 on the natural logarithmic scale (*p* < 0.001), corresponding to approximately 5,145 RMB after exponentiation. During the pre-DIP period, expenditures exhibited a statistically significant monthly increase of approximately 0.41% (*β*₁ = 0.0041, *p* = 0.032). The policy intervention did not result in a significant immediate level change or a notable shift in the post-policy trend (*β*₂ = −0.0100, *p* = 0.610; *β*₃ = 0.0019, *p* = 0.311).

**Table 5:** The changes in the diagnostic and therapeutic services expenditures per case.

Coefficient	Estimate	Standard error	*t*	*P*	95% confidence interval	Durbin-Watson statistic	Ljung–Box *P*-value
*β*_1_	0.00	0.00	2.23	0.032	0.00, 0.01	2.09	0.375
*β*_2_	−0.01	0.02	−0.51	0.610	−0.05, 0.03		
*β*_3_	0.00	0.00	1.03	0.311	0.00, 0.01		
*β*_0_	8.54	0.01	1023.80	0.000	8.53, 8.56		

#### 3.2.5 Changes in the OOP payments per case

**Figure 9:**
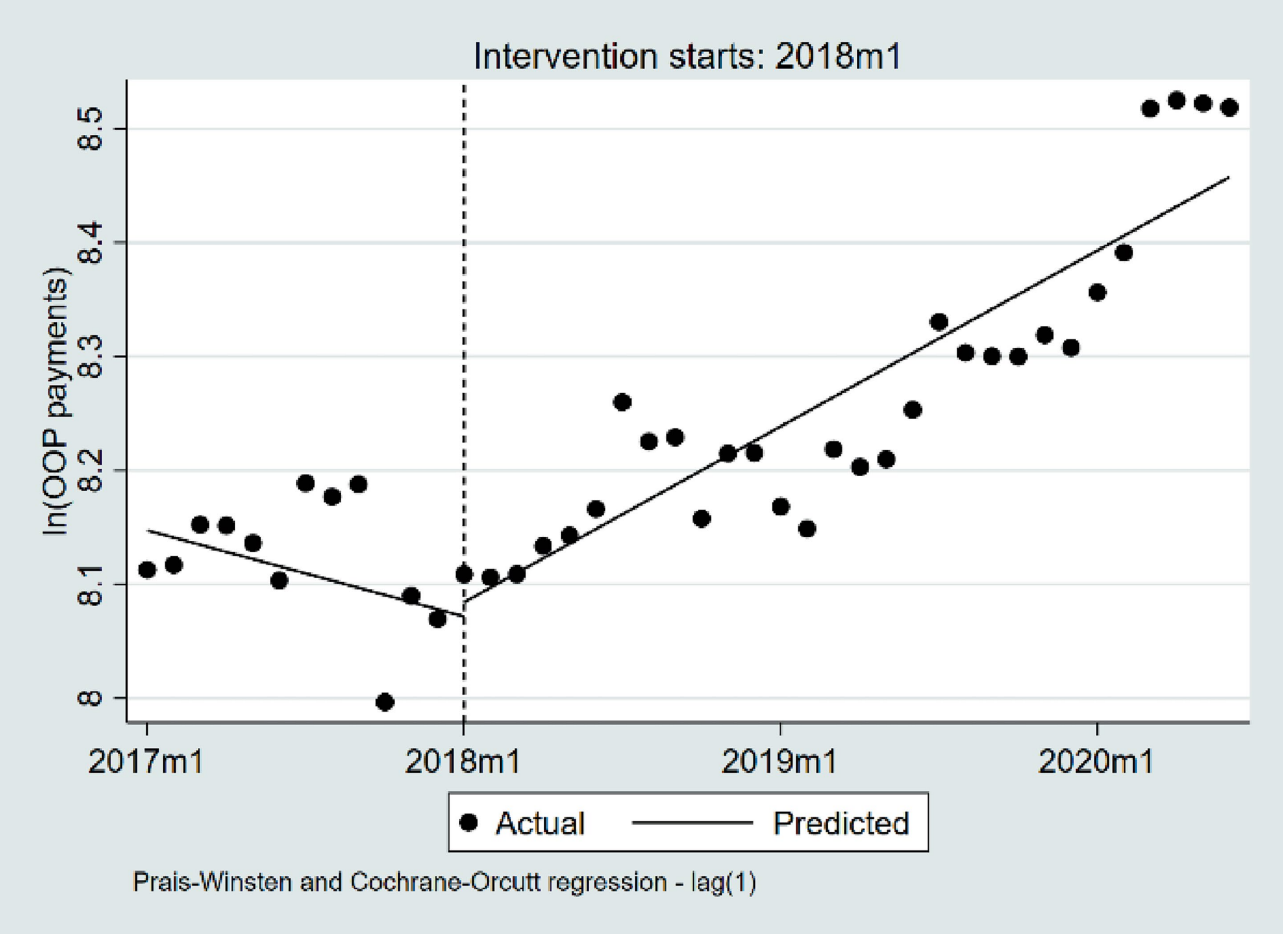
The changes in the OOP payments per case. OOP, out-of-pocket payments; 2017 m1, January 2017; 2018 m1, January 2018; 2019 m1, January 2019; 2020 m1, January 2020.

We summarize the changes in OOP payments per case in Table 6 and Figure 9. At the beginning of the observation period, the baseline level of OOP payments measured approximately 3,463 RMB (*p* < 0.001). During the pre-DIP period, OOP payments exhibited a nonsignificant downward trend, with an average monthly decrease of approximately 0.63% (*β*_1_ = −0.0063, *p* = 0.383). The implementation of the DIP policy was associated with a nonsignificant immediate level increase, corresponding to an estimated relative rise of approximately 1.25% (*β*_2_ = 0.0124, *p* = 0.832). Following the policy intervention, the monthly trend exhibited a statistically significant positive change relative to the pre-policy slope (*β*_3_ = 0.0192, *p* = 0.022), indicating an additional monthly increase of approximately 1.94% beyond the pre-existing trend.

**Table 6:** The changes in the OOP payments per case.

Coefficient	Estimate	Standard error	*t*	*P*	95% confidence interval	Durbin-Watson statistic	Ljung–Box *P*-value
*β*_1_	−0.01	0.01	−0.88	0.383	−0.02, 0.01	2.04	0.570
*β*_2_	0.01	0.06	0.21	0.832	−0.11, 0.13		
*β*_3_	0.02	0.01	2.39	0.022	0.00, 0.04		
*β*_0_	8.15	0.03	277.49	0.000	8.09, 8.21		

#### 3.2.6 Changes in the LOS

**Figure 10:**
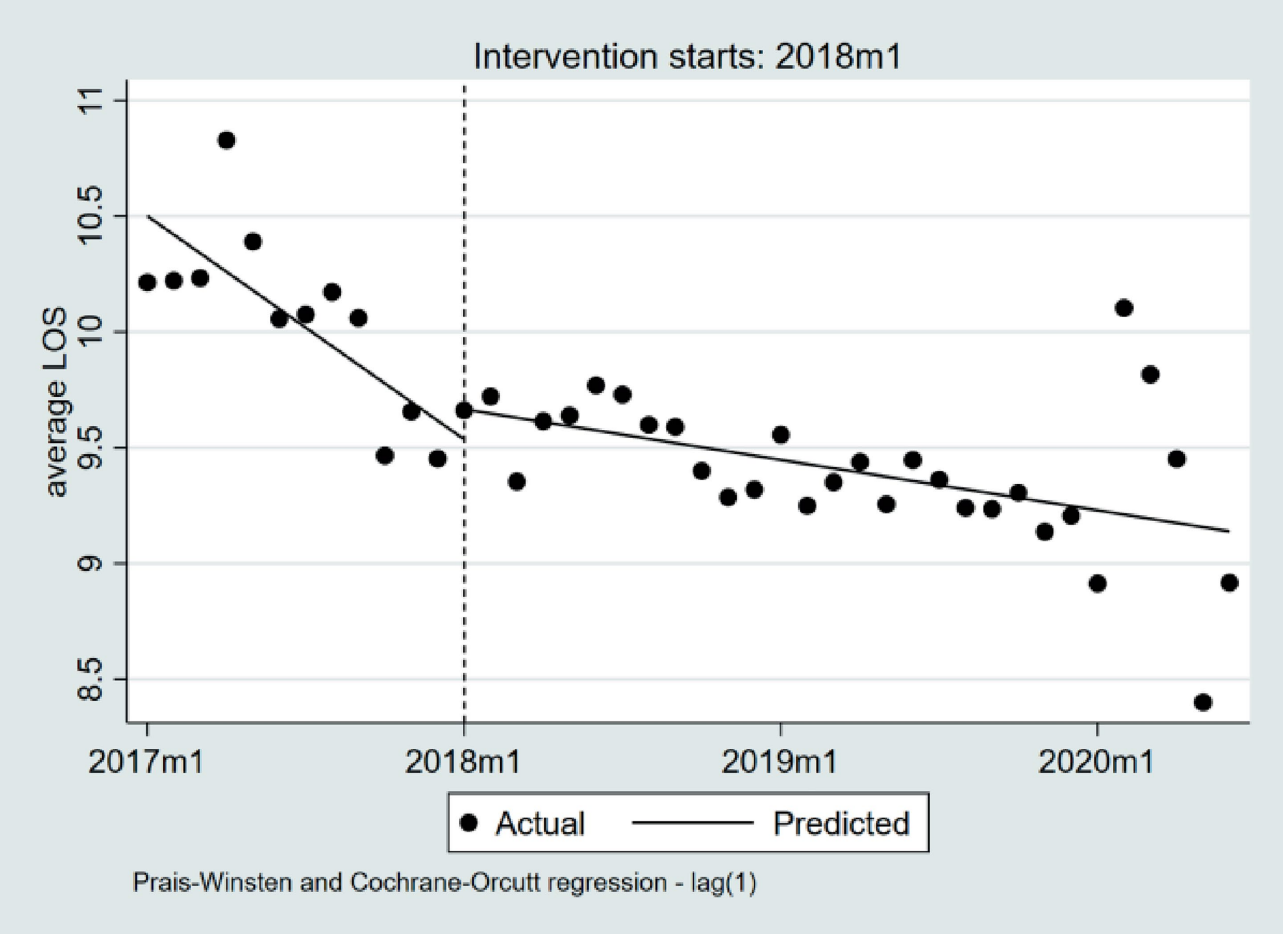
The changes in the LOS. LOS, length of stays; 2017 m1, January 2017; 2018 m1, January 2018; 2019 m1, January 2019; 2020 m1, January 2020.

Table 7 and Figure 10 presents the interrupted time series analysis of the average LOS per case. At baseline, the average LOS was estimated at 10.50 days (*p* < 0.001). During the pre-DIP period, a statistically significant downward trend was observed (*β*_1_ = −0.0804, *p* = 0.002), corresponding to a monthly reduction of approximately 0.08 days. At the implementation point of the DIP reform, the immediate level change was positive but not statistically significant (*β*_2_ = 0.1295, *p* = 0.449), corresponding to an increase of approximately 0.13 days. During the post-DIP period, the slope significantly increased relative to the pre-policy trend (*β*₃ = 0.0622, *p* = 0.022), indicating a slower rate of decline. The overall monthly reduction in LOS narrowed to approximately 0.018 days.

**Table 7:** The changes in the LOS.

Coefficient	Estimate	Standard error	*t*	*P*	95% confidence interval	Durbin-Watson statistic	Ljung–Box *P*-value
*β*_1_	−0.08	0.02	−3.38	0.002	−0.13, −0.03	1.87	0.395
*β*_2_	0.13	0.17	0.77	0.449	−0.21, 0.47		
*β*_3_	0.06	0.03	2.38	0.022	0.01, 0.12		
*β*_0_	10.50	0.18	58.06	0.000	10.13 10.87		

#### 3.2.7 Changes in the 30-day readmission rate

**Figure 11:**
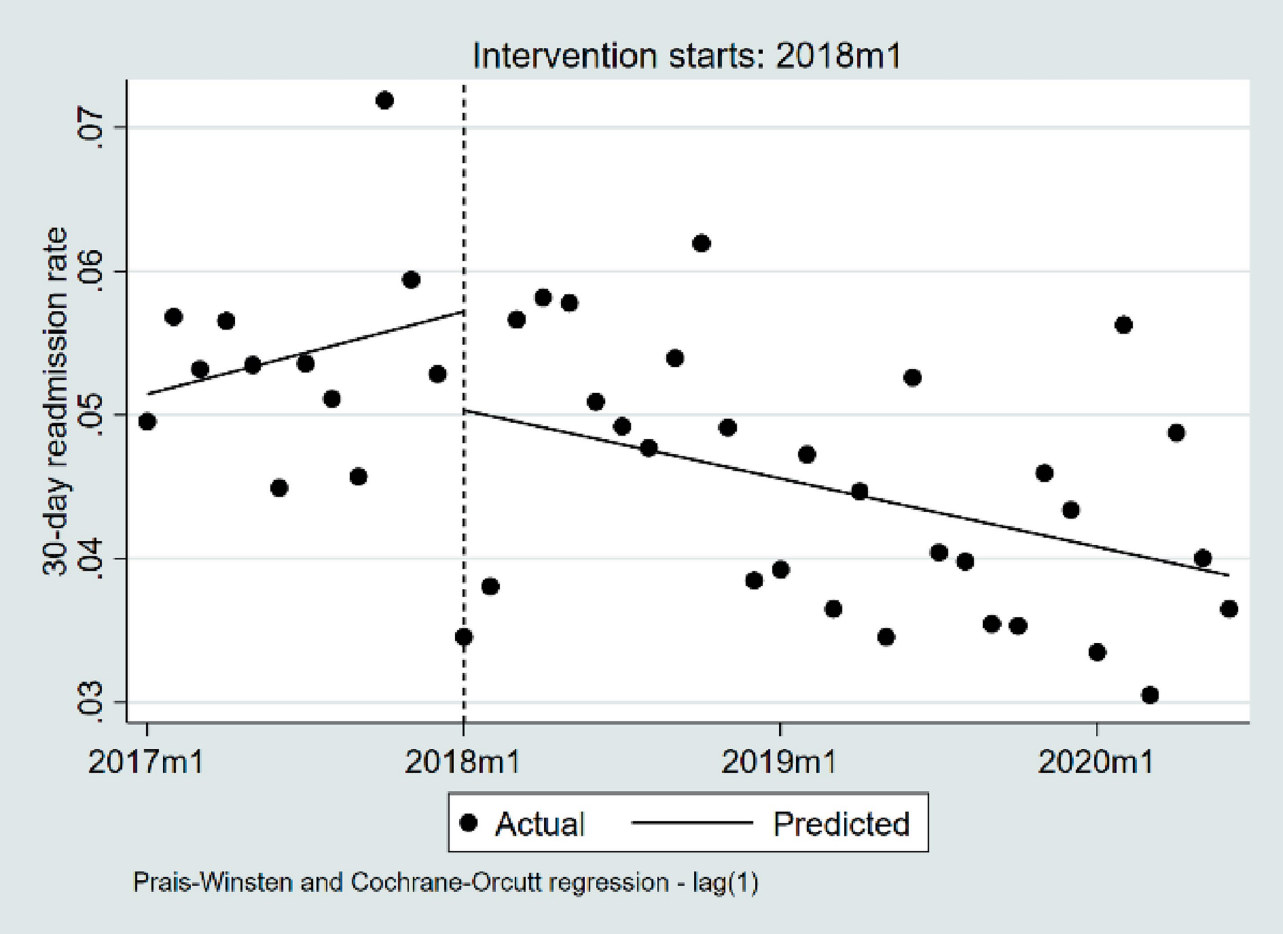
The changes in the 30-day readmission rate. 2017 m1, January 2017; 2018 m1, January 2018; 2019 m1, January 2019; 2020 m1, January 2020.

We summarize the changes in the 30-day readmission rate in Table 8 and Figure 11. At the beginning of the observation period, the baseline rate was 5.14% (*p* < 0.001). During the pre-DIP period, the rate exhibited a nonsignificant monthly increase of 0.05% (*β*₁ = 0.0005, *p* = 0.367). The implementation of the DIP policy was associated with a nonsignificant immediate decrease of 0.69% (*β*₂ = −0.0069, *p* = 0.262), and the post-policy monthly trend declined by an additional 0.09% (*β*₃ = −0.0009, *p* = 0.127), though this change was also not statistically significant, indicating that the DIP reform did not lead to significant deterioration in the 30-day readmission rate.

**Table 8:** The changes in the 30-day readmission rate.

Coefficient	Estimate	Standard error	*t*	*P*	95% confidence interval	Durbin-Watson statistic	Ljung–Box *P*-value
*β*_1_	0.0005	0.0005	0.9100	0.367	−0.0006, 0.0015	2.00	0.362
*β*_2_	−0.0069	0.0060	−1.1400	0.262	−0.0191, 0.0054		
*β*_3_	−0.0009	0.0006	−1.5600	0.127	−0.0020, 0.0003		
*β*_0_	0.0514	0.0022	23.9200	0.000	0.0471, 0.0558		

## 4 Discussion

Evaluating the effectiveness of the DIP payment system is a complicated issue. The DIP payment system is similar to the DRG payment system, with the primary difference being the classification of disease groups. As such, evaluating the effectiveness of the DIP payment reform using the same metrics used to assess the effectiveness of the DRG payment system is worthwhile.

In theory, the DIP payment system should improve the efficiency of healthcare efficiency and reduce patient financial burdens. Previous international studies provide empirical support. For instance, transitions from FFS to DRG systems in South Korea significantly reduced patients’ medical expenditures (22). Research from Switzerland reported comparable clinical outcomes between DRG and FFS models, highlighting potential moral hazards under FFS (23). In Taiwan, employing the DRG payment system effectively reduced the length of hospital stay without compromising patient treatment quality (24). Similarly, DRG-like case-based payment systems, such as Japan’s Diagnosis Procedure Combination/Per-Diem Payment Scale (DPC/PDPS), have demonstrated advantages in reducing patient financial burdens and shortening hospital stays compared to FFS (25). Moreover, studies within China consistently demonstrate the cost-reduction and efficiency-enhancement advantages of DRG-based systems relative to FFS (26–29).

However, our analysis of the DIP payment reform implemented in Guangzhou did not yield findings consistent with previously reported positive outcomes. While the average LOS continued to decline and the 30-day readmission rate remained stable, these trends suggest that the reform did not compromise care quality. When we focus on expenditure-related indicators, total inpatient costs and OOP payments both increased significantly, indicating that the DIP reform fell short in alleviating patients’ financial burden and containing hospital expenditures.

Our study investigates the underlying reasons why the DIP payment reform in Guangzhou has failed to reduce inpatient costs for patients, with particular attention to the incentives embedded in the DIP payment mechanism and the structure of its institutional design. As showed in Figure 2, hospital reimbursement under the DIP system is jointly determined by the RW assigned to each DIP group and the year-end point rate. Since RW values are recalculated annually based on historical inpatient cost averages, hospitals have a strong incentive to maintain or raise reported costs in order to preserve or increase future RW levels—so long as these costs remain within the designated outlier range. This incentive structure is consistent with our empirical findings, which show a sustained increase in inpatient cost following the implementation of the DIP reform.

However, while hospitals are motivated to increase inpatient cost to maintain RW, they must do so within the financial constraints imposed by fixed reimbursement rules. This encourages a marginal cost logic—favoring cost items that allow cost inflation without significant budgetary pressure. Such cost-driven behavior was further reinforced by subsequent policy changes, most notably the introduction of zero-markup policies, which transformed medications and medical consumables from profit sources into pure cost items. In the sample region of this study, local authorities introduced a zero-markup drug policy in July 2017 and extended it to consumables by the end of 2018, requiring hospitals to sell these items strictly at procurement price. Consequently, hospitals could no longer rely on these categories for revenue, turning instead to diagnostic and therapeutic services as primary sources of surplus — especially because physician salaries in China are largely fixed, and service pricing does not tightly correspond to delivery cost. This structural shift enabled hospitals to leverage low-cost services to sustain revenue flows.

Our empirical results support this strategic shift. The average medication expenditures per case declined significantly prior to the DIP reform—likely reflecting the effect of the zero-markup policy—but stabilized afterward. The cost of consumables showed no notable change across the observed phases. In contrast, expenditures on diagnostic and therapeutic services exhibited a sustained upward trend throughout the study period, and our analysis suggests that the implementation of the DIP reform did not produce a significant moderating effect on this growth. These patterns are consistent with our hypothesis regarding hospital profit-maximization behavior under the DIP framework.

Building on the preceding analysis, we further investigate the mechanisms underlying the observed increase in patients’ OOP payments following the DIP reform. A central mechanism involves the year-end point rate calculation, which determines the monetary value assigned to each RW and thereby scales the final reimbursement standard for each DIP group. As showed in Stage 3 of Figure 2, the year-end point rate is calculated by dividing the region global budget by the actual reimbursement ratio at the city level—defined as the share of inpatient cost paid by the medical insurance fund, then divided by the aggregate number of RW_i_ by all hospitals in the region. As a result, the year-end point rate is inversely correlated with the reimbursement ratio: a lower reimbursement ratio leads to a higher year-end point rate. This inverse relationship creates an implicit incentive for hospitals to increase patients’ OOP payments, since doing so reduces the reimbursement ratio and, in turn, raises the year-end point rate applied to submitted RW values—ultimately increasing overall reimbursement.

Secondly, the rise in OOP payments is also closely linked to the method used in Guangzhou for calculating the final reimbursement amount received by hospitals. As illustrated in Stage 4 of Figure 2, hospital’s income under the DIP system consists of two components: (1) the OOP payments made by patients, and (2) the reimbursable portion of the PSᵢ, which is calculated as the product of the hospital’s RWᵢ and the year-end point rate. It is essential to note that the PSᵢ reflects a theoretical benchmark for total inpatient expenditure, rather than the actual amount to be paid by the insurance fund. To determine the actual insurance payment, it is necessary to deduct the portion not covered by the insurance fund. Guangzhou uses the multiplication method to estimate this deductible portion by directly applying the hospital’s reimbursement ratio to PSᵢ, yielding a theoretical insurance payout. By contrast, some other regions use the subtraction method, which simply deducts the actual OOP amount from PSᵢ.

While both methods aim to reflect the division of financial responsibility between the patient and the insurer, they differ significantly in their financial implications. Specifically, when a hospital operates at a DIP deficit—that is, its actual inpatient costs exceed the assigned PSᵢ—the multiplication method tends to benefit the hospital. This is because the actual OOP payments collected from patients often exceed the OOP share implied by the multiplication method. In effect, hospitals are able to partially shift financial pressure from themselves to the insurance fund and patients by encouraging or allowing higher OOP charges. Conversely, when a hospital’s actual costs are below PSᵢ (i.e., operating at a DIP surplus), raising OOP payments leads to a larger deduction from PSᵢ, thus reducing the hospital’s income under the multiplication method. However, since hospitals in surplus are already operating above their breakeven point, they may be less sensitive to this marginal income loss.

Taken together, this asymmetric reimbursement logic under the multiplication method introduces a built-in incentive for deficit-operating hospitals to raise OOP payments, even in the absence of explicit price hikes. Drawing on prospect theory from behavioral economics, hospitals may exhibit loss aversion, being more motivated to avoid deficits than to increase surpluses. This behavioral response helps explain the observed post-reform rise in patient financial burden in Guangzhou.

Moreover, because China’s DIP system derives RW from historical inpatient cost rather than actual treatment costs, hospitals reporting deficits under DIP may not necessarily be incurring real financial losses. The absence of transparent and standardized cost-accounting systems obscures the true relationship between service delivery and cost recovery, granting providers substantial discretion to adjust OOP payments as a tool for revenue balancing. This institutional opacity not only weakens accountability in cost control but also creates fertile ground for moral hazard. As demonstrated by our empirical findings, hospitals may strategically restructure service components or elevate OOP payments to optimize reimbursement under conditions of informational asymmetry and insufficient regulatory oversight. Furthermore, since OOP payments are typically collected directly from patients at discharge, they enhance hospital liquidity and provide a buffer against short-term cash flow constraints, further reinforcing the financial incentive to shift unreimbursed costs onto patients.

Accordingly, in designing medical insurance payment mechanisms, policymakers should carefully consider how algorithmic choices affect provider incentives, cost-sharing distribution, and the equitable use of insurance funds.

While our findings offer valuable insights for policy refinement, several limitations of this study should be acknowledged. First, due to the nature of administrative data, important individual-level variables such as income, employment status, and comorbidities were unavailable, which may affect the interpretation of results. Second, the analysis focused primarily on cost-related outcomes, without incorporating direct measures of care quality or provider behavior. Third, the study was conducted in Guangzhou, a relatively affluent region, which may limit the generalizability of findings to other settings. Fourth, the lack of a control group restricts our ability to make strong causal claims. Although we used Interrupted Time Series Analysis to address this issue, future research should consider using methods such as Difference-in-Differences or matched comparisons to improve causal inference.

## 5 Conclusion

The results indicated that the DIP payment reform implemented in Guangzhou did not fully achieve its intended objectives of containing inpatient costs and alleviating patients’ financial burdens. Our analysis attributes these unexpected outcomes to institutional incentives embedded in the DIP payment system, particularly its reliance on historical inpatient cost to set reimbursement standards, coupled with the profit-driven behaviors of hospitals.

The findings of this study offer important implications for healthcare financing policy in China and other developing nations. This research contributes to the existing body of knowledge regarding prospective payment systems, highlighting both the feasibility and complexity of implementing DRG-like reforms in developing country contexts. Specifically, this study provides detailed insights into how certain algorithmic choices within DIP, such as RW calculations and reimbursement deduction methods, can inadvertently increase patients’ financial burdens. Thus, our findings offer valuable considerations for future policy optimization within the medical insurance sector.

Compared to existing literature (30–33), this study not only conducted an empirical evaluation of the DIP reform but also, to the best of our knowledge, is the first to explore the underlying mechanisms through which reimbursement algorithms influence hospital behavior and patient financial burden, thereby addressing a critical gap in current research. Looking ahead, future studies will seek to incorporate richer datasets and adopt perspectives from organizational behavior and comparative policy analysis to further deepen understanding of the reform’s long-term effects and institutional dynamics.

## Data Availability

The raw data supporting the conclusions of this article will be made available by the authors, without undue reservation.
